# Mindful parenting, parental involvement, and preschoolers’ emotional competence in parent-grandparent co-parenting families in China: an explanatory sequential mixed-methods study

**DOI:** 10.1186/s41155-026-00409-x

**Published:** 2026-08-18

**Authors:** Dongyu Wang, Tongyao Wang, Ruchuan Zhou, Peiyue Wu

**Affiliations:** 1 Faculty of Education, Qufu Normal University, Qufu, China; 2 School of Data and Computer Science, Shandong Women’s University, Jinan, China; 3 School of Education, Central China Normal University, Wuhan, China; 4 School of Marxism, Dongguan Polytechnic, Dongguan, China; 5 School of Psychology, Central China Normal University, Wuhan, China

**Keywords:** Emotional competence, Parent-grandparent co-parenting, Mindful parenting, Parental involvement, Grandparent support, Family ecology, Mixed methods, Network analysis

## Abstract

**Background:**

Emotional competence is central to preschoolers’ socioemotional development and is embedded in everyday family interactions. However, relatively little is known about how parenting processes operate in parent-grandparent co-parenting contexts in China. Guided by Belsky’s process model of parenting, this study examined how mindful parenting and parental involvement were associated with family resources, intergenerational caregiving, and preschoolers’ emotional competence within parent-grandparent co-parenting families.

**Methods:**

This study used an explanatory sequential mixed-methods design. In the quantitative phase, 4,020 parents from parent-grandparent co-parenting families in 40 kindergartens across eight provinces in China completed questionnaires. Hierarchical regression and Gaussian graphical network analysis were used to examine associations among parent characteristics, family resources, child characteristics, mindful parenting, parental involvement, and preschoolers’ emotional competence. In the qualitative phase, semi-structured interviews with 20 parents were analyzed using constructivist grounded theory.

**Results:**

Quantitative results showed that mindful parenting, parental involvement, and grandparent support were positively associated with preschoolers’ emotional competence in the final regression model, whereas parental depression and child social withdrawal were negatively associated with it. In the network analysis, home-based parental involvement, mindful discipline, and grandparent support had relatively prominent positive bridge expected-influence point estimates, but these estimates did not differ significantly from one another. Qualitative findings elaborated how parents described emotional regulation, mindful responses, everyday involvement, intergenerational coordination, and adjustments to children’s behavior as interconnected aspects of family emotional caregiving.

**Conclusions:**

The findings highlight an associative parent-grandparent co-parenting ecology in which mindful parenting and parental involvement were related to family resources, intergenerational coordination, and preschoolers’ emotional competence. Efforts to support preschoolers’ emotional competence may therefore consider not only the extent of parental involvement, but also the emotional quality of parenting and the coordination of caregiving between parents and grandparents.

**Supplementary Information:**

The online version contains supplementary material available at https://doi.org/10.1186/s41155-026-00409-x.

## Introduction

Emotional competence involves children’s developing skills in recognizing, interpreting, communicating, and managing emotions in both themselves and other people (Halle & Darling-Churchill, 2016). It not only shapes the quality of children’s interactions with parents, teachers, and peers, but also lays the foundation for later school readiness, social adjustment, and mental health (Denham, 2006; Denham et al., 2003). Because preschoolers’ regulatory capacities are still developing, their emotional learning is strongly shaped by the responsiveness, modeling, and guidance available in daily caregiving relationships (Hajal & Paley, 2020; Morris et al., 2007). In Chinese families in particular, young children’s daily care is often provided not only by parents but also by grandparents, who are frequently deeply involved in childcare. This caregiving arrangement is associated with intergenerational reciprocity, family obligation, and the traditional value of filial piety, as well as with parental employment pressures, the allocation of family resources, and multigenerational household structures (Chen et al., 2011). Thus, parent-grandparent co-parenting provides a culturally salient caregiving context for examining preschoolers’ emotional competence in China. In this context, children’s emotional experiences are shaped not only by parent-child interactions but also by intergenerational caregiving coordination, consistency in discipline, and the ways parents transform grandparent support into emotionally responsive involvement. Examining these processes can extend child development research by foregrounding intergenerational co-caregiving in a non-Western family context (Raval & Walker, 2019).

Two family processes are especially relevant for understanding this issue: the emotional quality of parents’ responses, captured by mindful parenting, and the practical forms of family engagement, captured by parental involvement. Mindful parenting describes parents’ capacity to stay attentive during interactions with children, notice children’s emotional signals without immediate judgment, and respond with emotional regulation and flexibility (Duncan et al., 2009). It comprises two dimensions, being in the moment with the child and mindful discipline, and represents parents’ proximal regulatory resources when responding to children’s emotional and behavioral cues (McCaffrey et al., 2017). Parental involvement concerns parents’ engagement in children’s learning and development across family and kindergarten settings, including communication with teachers, participation in kindergarten activities, and developmentally supportive activities at home. It captures the practical forms through which family caregiving processes are enacted in daily life and in home-kindergarten collaboration (Fantuzzo et al., 2000). Together, these two constructs highlight regulatory responsiveness and concrete involvement processes in family caregiving, illustrating how parents provide emotional support and developmental opportunities for children through everyday interactions and home-kindergarten coordination (Cosso et al., 2022; McKee et al., 2018).

Family socioeconomic resources and marital relationships not only shape the educational and caregiving resources available to the family, but may also influence the emotional support children receive in daily life through economic stress, coparental coordination, and the broader family emotional climate (Conger & Donnellan, 2007; Teubert & Pinquart, 2010). This makes parent-grandparent co-parenting theoretically important because grandparental involvement may operate both as a source of caregiving support and as a context in which parents must negotiate emotional responses, rules, and educational beliefs across generations (Chen et al., 2011; Xu et al., 2024). At the same time, pressures associated with modern life and the use of digital media are reshaping the quality of parent-child interactions (McDaniel, 2019). Parental depression may weaken parents’ sensitive responses to children’s emotions, whereas parental phubbing may disrupt shared attention and immediate responsiveness between parents and children (Lovejoy et al., 2000; Vanden Abeele et al., 2020). From this perspective, young children’s emotional competence is not shaped by any single family factor; rather, it develops within a daily ecology in which resource conditions, intergenerational caregiving, parents’ psychological states, digitalized interactions, and children’s own social behaviors are intertwined. However, existing studies have often examined mindful parenting, parental involvement, family resources, or parents’ psychological and behavioral characteristics in relation to child development separately, with relatively little work comparing their relative contributions and interconnections within a single framework (Taraban & Shaw, 2018). A broader analytic perspective is therefore needed to examine these family, parent, and child factors simultaneously and to clarify how they are interrelated rather than considering them as separate predictors (Epskamp et al., 2018). Such integration is particularly important in the context of Chinese preschool families (Wang et al., 2026).

To address this gap, the present study uses Belsky’s process model of parenting as an organizing framework for understanding the relations among multiple levels of family, parent, and child factors, rather than treating these factors as a list of independent predictors. In this model, parenting is situated at the intersection of parent characteristics, child characteristics, and broader contextual sources of support and stress (Belsky, 1984; Taraban & Shaw, 2018). Accordingly, family SES, marital satisfaction, and grandparent support are conceptualized as contextual conditions that may be associated with the resources available for caregiving. Parental depression and parental phubbing represent parents’ psychological and attentional conditions that may be associated with their capacity for attentive and responsive interaction. Children’s social withdrawal represents a child characteristic that may be associated with opportunities for emotional interaction as well as with how parents respond and become involved. Mindful parenting and parental involvement are conceptualized as complementary proximal parenting processes: mindful parenting captures the attentional and regulatory quality of parental responses, whereas parental involvement captures how caregiving is enacted through home-based activities, kindergarten participation, and home-kindergarten communication.

These domains are therefore expected to be interrelated rather than to operate as isolated influences. Contextual resources may be connected with parents’ psychological functioning and the support available for caregiving; parental psychological and attentional conditions may be associated with mindful parenting and parental involvement; and child characteristics may be related to both children’s emotional competence and parents’ opportunities for interaction. The present study examines this theoretical organization at two complementary levels: hierarchical regression assesses construct-level associations with children’s overall emotional competence, whereas network analysis describes dimension-level conditional associations and cross-community connections. Because the data are cross-sectional, this framework specifies the theoretical organization of the variables but does not assume or test temporal, mediating, or causal pathways.

## Literature review

Against this background, mindful parenting represents one of the two proximal parenting processes examined in the present study. Previous studies indicate that mindful parents tend to be more attentive to children’s emotional signals, less reactive in discipline, and more likely to use supportive emotion-socialization practices (McKee et al., 2018). Longitudinal evidence further indicates that mindful parenting predicts the subsequent development of children’s emotional self-regulation (Quach et al., 2026). At the same time, mindful parenting is closely associated with parental stress, positive parenting practices, and the quality of the parent-child relationship (Coatsworth et al., 2015; Parent et al., 2020). Children’s emotional competence has also been associated with broader patterns of parenting, particularly authoritative parenting (Mortazavizadeh et al., 2022). Taken together, these findings suggest that mindful parenting is positively associated with children’s emotional competence and is also embedded within the broader family caregiving system. Accordingly, the present study proposes Hypothesis 1: *Mindful parenting is significantly and positively associated with young children’s emotional competence*,* and its dimensions are expected to occupy positive cross-community connecting positions in the network.*

Parental involvement captures the ways parents take part in children’s development through everyday family activities and collaboration with kindergarten settings. Fantuzzo et al. (2000) distinguished parental involvement in early childhood education into home-kindergarten communication, kindergarten-based involvement, and home-based involvement, indicating that parental involvement is not limited to school activities but also occurs through family interactions, home-kindergarten information exchange, and collaboration with educational settings. Parent-child interaction, shared activities, rule negotiation, and home-kindergarten communication embedded in parental involvement provide important contexts for the development of children’s emotional competence (Fantuzzo et al., 2004). Parental involvement has also been shown to enhance children’s social-emotional competence (Cosso et al., 2022). Evidence from intervention research also suggests that strengthening responsive parenting may support the growth of emotional competence in young children (England-Mason et al., 2023). In addition, home-school communication and parent-teacher trust are associated with the development of young children’s social-emotional competence (Hummel et al., 2023; Sheridan et al., 2019). However, the present study did not directly assess teacher characteristics, practices, or perceptions. Teachers were therefore not modeled as independent predictors or mediators. Instead, communication with teachers was treated as one component of parental involvement through which parents obtained information about their children’s kindergarten experiences and coordinated subsequent support at home (Fantuzzo et al., 2004). At the same time, parental involvement does not occur in isolation; rather, it is jointly shaped by family resources and parents’ psychological resources. Family socioeconomic status may affect the time and resources parents can devote to companionship, communication, and home education, while parental stress and the quality of the parent-child relationship may also influence the stability of parental involvement and the quality of parental responsiveness. Accordingly, the present study proposes Hypothesis 2: *Parental involvement is significantly and positively associated with young children’s emotional competence*,* and its dimensions are expected to occupy positive cross-community connecting positions in the network.*

From the perspective of Belsky’s model, family resources form broader conditions under which parenting practices are organized and enacted (Belsky, 1984; Taraban & Shaw, 2018). The present study focuses on three aspects: socioeconomic status (SES), marital satisfaction, and grandparent support. SES may influence the material resources, informational resources, and developmental opportunities available to families through parents’ education, occupation, and income, thereby further shaping the development of children’s emotional competence (Bradley & Corwyn, 2002; Conger & Donnellan, 2007). Marital satisfaction may affect parent-child interactions through a spillover pathway, whereby the emotional climate and conflict within the marital relationship extend into parents’ interactions with their children (Kouros et al., 2014). Finally, although grandparental caregiving may, in some contexts, give rise to intergenerational conflict over childrearing (Chen et al., 2011; Song & Zhang, 2012), recent findings indicate that grandparents’ emotional support and routine caregiving may contribute positively to preschoolers’ socioemotional development (Xu et al., 2024). Thus, grandparent support is not only a background family resource, but also a co-parenting condition that may shape how parents regulate emotions, maintain involvement, and coordinate children’s daily emotional socialization across generations. Accordingly, the present study proposes Hypothesis 3: *SES*,* marital satisfaction*,* and grandparent support within parent-grandparent co-parenting are significantly and positively associated with young children’s emotional competence.*

Parent characteristics constitute another core domain in the process model of parenting. In the present study, parental depression and parental phubbing represent two types of parent characteristics that may constrain the development of young children’s emotional competence: the former primarily concerns parents’ emotional states and regulatory resources, whereas the latter reflects an interactional context in which parents’ attention is diverted by mobile devices in digitalized family life. When parents experience depression, low energy, or high stress, their own emotional experiences and emotion-regulation capacities may be constrained, making it more difficult for them to provide stable, sensitive, and supportive emotional responses (Hajal & Paley, 2020). Empirical research has shown that maternal depressive symptoms are associated with children’s socioemotional reactivity (Premo & Kiel, 2016). Parental phubbing reflects another form of risk in contemporary digital family contexts. According to technoference theory, parents’ excessive attention to smartphones during parent-child interactions may disrupt shared attention and immediate responsiveness, reduce the continuity and sensitivity of interactions, and be associated with children’s internalizing and externalizing behavior problems (McDaniel & Radesky, 2018). Further research suggests that parental phubbing may be linked to children’s social withdrawal through parent-child conflict and children’s negative emotions (Zhang & Wang, 2025). Accordingly, the present study proposes Hypothesis 4: *Parental depression and parental phubbing are significantly and negatively associated with young children’s emotional competence.*

At the child level, this study also considers social withdrawal as a behavioral characteristic relevant to emotional competence. Social withdrawal is manifested as a preference for solitude, social anxiety, or active avoidance, and represents an important behavioral feature of socioemotional development in preschool children. Developmental research has shown that socially withdrawn children are more likely to experience peer difficulties, internalizing problems, and risks in social adjustment (Rubin et al., 2009), and may even have fewer opportunities to develop emotion knowledge and broader emotional competence (Clark et al., 2022). Accordingly, the present study proposes Hypothesis 5: C*hildren’s social withdrawal is significantly and negatively associated with young children’s emotional competence.*

Building on this integrative framework, the study investigated how family resources, parent characteristics, child characteristics, mindful parenting, and parental involvement were related to emotional competence among Chinese preschoolers in parent-grandparent co-parenting contexts. Specifically, we tested whether mindful parenting and parental involvement were associated with young children’s emotional competence and compared these associations with those involving family resources, parent characteristics, and children’s social withdrawal. We further examined how these factors were organized within an associative network and identified variables that played central or bridging roles across parent characteristics, family resources, parenting processes, and child outcomes. Finally, we used semi-structured interviews to elaborate how parents described and understood these links in everyday family life.

## Method

### Participants

For the quantitative phase, the sample consisted of parents of preschool-aged children drawn from 40 kindergartens located in eight Chinese provinces. The participating kindergartens were distributed across northern, southern, eastern, central, and western regions, which increased the geographical breadth of the sample. Respondents were required to be the child’s primary caregiver, and the final analytic sample was restricted to parents of children enrolled in preschool settings. Eligibility additionally required that at least one grandparent regularly participated in the child’s care or provided ongoing childrearing support. Co-residence with grandparents was not required. Following data screening, 4,020 questionnaires met the inclusion criteria and were included in the analyses. Respondents had a mean age of 35.23 years (*SD* = 4.60, range = 21–55); 80.52% were mothers or female caregivers, whereas 19.48% were fathers or male caregivers. Children had a mean age of 53.62 months (*SD* = 12.63, range = 24–83), and 53.13% were boys. Participants covered all kindergarten class levels, including lower class (31.32%), middle class (32.44%), upper class (32.49%), and other class levels (3.76%). Family SES was derived through factor analysis based on parental education, occupational status, and monthly household income; detailed coding and score-construction procedures are provided in Supplementary Appendix A. The analysis yielded one factor with an eigenvalue greater than 1, accounting for 65.70% of the variance; higher factor scores represented higher SES. Additional demographic characteristics are presented in Supplementary Table S1.

For the qualitative phase, interviewees were selected purposively to ensure variation in parental education, occupation, age, and caregiving coordination, especially in relation to intergenerational caregiving. All interview participants reported ongoing grandparental involvement in childcare or childrearing support, although grandparents were not necessarily co-resident with the child. Altogether, 20 parents participated in interviews, producing 816 min of audio data and 98,454 words of transcripts. Interview participant characteristics are summarized in Supplementary Table S2. The study protocol was reviewed and approved by the ethics committee of the first author’s institution, and all participants provided informed consent before participation.

### Procedure

An explanatory sequential mixed-methods design was used: the survey phase was completed first, followed by an interview phase designed to contextualize and elaborate the quantitative patterns (Creswell & Plano Clark, 2017). In the first phase, guided by the process model of parenting, the study examined the associations of mindful parenting, parental involvement, family resources, parent characteristics, and child characteristics with young children’s emotional competence. After providing informed consent, parents completed the anonymous online questionnaire voluntarily. In the second phase, semi-structured interviews were conducted to complement and interpret the quantitative findings, with a focus on how parents understood young children’s emotions and described the relationships among mindful parenting, parental involvement, family resources, and children’s emotional competence in everyday family life (Charmaz, 2006). A total of 20 parents were interviewed individually in face-to-face sessions. The interview protocol addressed caregiving coordination, family resources, parents’ emotional states and parenting experiences, mindful parenting practices, parental involvement, children’s emotional expression and regulation, and parents’ interpretations of intergenerational family influences. All quantitative and qualitative data were provided by parents. Children’s emotional competence and social withdrawal were assessed through parent reports, while references to teachers and grandparents in the qualitative findings represented parents’ perceptions and accounts rather than independently assessed stakeholder perspectives.

## Measures

### Children’s emotional competence

Children’s emotional competence was measured with the Children’s Emotional Competence Development Scale, developed by Jian (2008) and later revised by Xu (2023). The revised scale includes 13 items covering emotional understanding, emotional regulation, emotional expression, and emotional awareness. Responses were scored using a five-category response format, and mean scores were calculated for both the total scale and its subscales. Higher mean scores reflected stronger emotional competence. Reliability in this sample was satisfactory, with Cronbach’s α values of 0.896 for the total scale and 0.783, 0.742, 0.717, and 0.736 for the four subscales, respectively.

### Mindful parenting

Mindful parenting was measured with the Chinese version of the Mindfulness in Parenting Questionnaire (MIPQ), adapted by Zhao (2018) from McCaffrey et al. (2017). The instrument contains 28 items scored on a 4-point Likert scale from 1= occasionally to 4 = always. Two subscales are included: mindful discipline and present-moment attention. Mindful discipline captures parent-focused regulatory qualities, such as nonreactivity, parenting awareness, and goal-directed responses. Present-moment attention reflects child-focused qualities, including present-focused attention, empathy, and acceptance of the child. Higher scores indicate more frequent mindful parenting behavior. In the present sample, Cronbach’s *α* was 0.958 for the total scale and 0.907 and 0.963 for mindful discipline and present-moment attention, respectively. McDonald’s ω was 0.957 for the total scale and 0.904 and 0.963 for the two subscales, respectively. In the present study, the MIPQ operationalized mindful parenting as parents’ self-reported frequency of mindful parenting behaviors; it did not constitute a direct observational assessment of parent-child interaction.

### Parental involvement

Parental involvement was measured with the Chinese short form of the Parental Involvement Questionnaire, adapted from Fantuzzo et al. (2000) and revised for Chinese preschool parents by Liu and Li (2019). This 20-item instrument assesses three forms of involvement: communication between home and kindergarten, participation in kindergarten activities, and involvement in home-based activities. Parents responded on a 4-point scale from 1 = rarely to 4 = always. Higher scores represented higher levels of parental involvement. The total scale demonstrated strong reliability in this sample, with Cronbach’s α = 0.953; the three subscales showed α values of 0.957, 0.893, and 0.914.

### Marital satisfaction

Marital satisfaction was measured with the Marital Satisfaction Subscale, which was developed by Fowers and Olson (1993) and adapted into Chinese by Wang et al. (1999). The subscale includes 10 items scored on a 5-point Likert scale, including five reverse-coded items. Higher scores reflected greater satisfaction with the respondent’s marital relationship. Reliability was acceptable in the present sample, with Cronbach’s α = 0.899 and McDonald’s ω = 0.890.

### Grandparent support

Grandparent support within parent-grandparent co-parenting was measured using the parenting-support subscale from the Grandparents-Parents Co-parenting Relationships Scale, based on Feinberg (2003) and revised by Li et al. (2016). The six items were scored on a 7-point scale from 1 = completely disagree to 7 = completely agree. Higher mean scores indicated stronger perceived support from grandparents in childrearing. The subscale showed high reliability, with both Cronbach’s α and McDonald’s ω equal to 0.952.

### Parental depression

Parental depressive symptoms were assessed with the Chinese short form of the Center for Epidemiologic Studies Depression Scale (CES-D-10) (Radloff, 1977), as adapted and validated in Chinese by He et al. (2013). The CES-D-10 includes 10 items, two of which were reverse scored. Responses were scored on a 4-point format, and the analyses used the mean item score. Higher values represent more severe depressive symptoms. The reliability estimates in this sample were 0.793 for Cronbach’s α and 0.739 for McDonald’s ω.

### Parental phubbing

Parental phubbing was measured with Zhang and Zhang’s (2020) revised version of the scale developed by Chotpitayasunondh and Douglas (2018). The scale contains 15 items representing four dimensions: phone separation anxiety, interpersonal conflict, self-isolation, and problem recognition. Items were scored on a 7-point Likert scale; higher scores reflected more frequent phubbing behavior. In the present sample, Cronbach’s *α* was 0.919 for the total scale and 0.867, 0.849, 0.913, and 0.812 for phone separation anxiety, interpersonal conflict, self-isolation, and problem recognition, respectively. McDonald’s ω was 0.914 for the total scale and 0.863, 0.849, 0.915, and 0.819 for the four subscales, respectively.

### Child social withdrawal

Child social withdrawal was assessed with the revised Chinese Child Social Preference Scale (Yin, 2023). The measure includes five items scored from 1 (completely disagree) to 5 (completely agree). Higher scores reflected a stronger tendency toward social withdrawal. In the present sample, Cronbach’s *α* was 0.733 and McDonald’s ω was 0.750.

## Data analyses

The quantitative analyses were conducted in SPSS 25.0 and R 4.4.3. Before the main analyses, potential common method bias was examined, followed by descriptive statistics and Pearson correlations for the study variables (Podsakoff et al., 2003). The two quantitative analyses were based on the same quantitative sample but addressed complementary and distinct research questions. Hierarchical regression was used to examine construct-level associations with children’s overall emotional competence while controlling for relevant covariates and to assess the incremental variance associated with theoretically ordered blocks of variables. Variables were entered sequentially in six blocks: demographic covariates, including parent age, parent gender, child age, and child gender; parental characteristics, including parental depression and parental phubbing; family resources, including SES, grandparent support, and marital satisfaction; child characteristics, including child social withdrawal; mindful parenting; and parental involvement.

Overall scale scores were used in the regression analyses to test construct-level hypotheses, whereas validated subscale scores were used as network nodes to examine dimension-specific associations; unidimensional constructs remained single nodes. Network analysis complemented the regression analysis by examining dimension-level conditional associations and cross-community connectivity. Before estimation, the network variables were residualized for parent age, parent gender, child age, and child gender. A Gaussian graphical model was estimated in the qgraph package using EBICglasso (γ = 0.50; threshold = TRUE), which was selected as a commonly used conservative setting that favors a sparser network and reduces the likelihood of retaining potentially spurious edges (Epskamp & Fried, 2018; Janková & van de Geer, 2018). One-step bridge expected influence was treated as the primary signed bridge-centrality index, whereas bridge strength was reported as a complementary descriptive index. Network accuracy and stability were evaluated using 1,000 nonparametric and 1,000 case-dropping bootstrap samples. Nonparametric bootstrapping was used to assess edge-weight accuracy and conduct pairwise difference tests for edge weights, node strength, expected influence, and bridge expected influence, whereas case-dropping bootstrapping was used to estimate correlation-stability coefficients. Neither quantitative analysis tested mediation, temporal ordering, or causal mechanisms. An exploratory respondent-gender Network Comparison Test is reported in Supplementary Appendix C.

To develop a contextually grounded account of how parents understood the family processes surrounding young children’s emotional competence, the qualitative interview data were analyzed using constructivist grounded theory. Belsky’s (1984) process model was used as a sensitizing framework rather than as a predetermined coding scheme or hypothesis-confirmation template (Charmaz, 2014). Accordingly, the analysis aimed to construct a substantive account of how family resources and parent-grandparent coordination, parent characteristics, mindful parenting, parental involvement, and child characteristics were connected in parents’ accounts, rather than to verify the model. Consistent with constructivist grounded theory, the analysis remained open to relationships, contradictions, and contextual processes not specified in the original model, including intergenerational negotiation, digitalized caregiving, home-kindergarten communication, and parents’ responses to child behavior (Charmaz, 2006). The qualitative analysis followed three iterative phases. In the first phase, initial coding was carried out independently by the principal investigator and a trained graduate researcher, who reviewed the transcripts line by line and recorded analytic memos. They then compared their coding decisions, discussed differences, and revised the preliminary codebook through analytic consensus. In the second phase, focused coding was used to consolidate the initial codes and develop broader conceptual categories. Category development was guided by constant comparison across participants with different demographic and caregiving backgrounds. As categories emerged, subsequent participant selection and interview prompts were adjusted to elaborate underdeveloped categories and examine contrasting caregiving configurations, consistent with theoretical sampling. Data collection continued until later interviews yielded no substantively new categories. In the final phase, all transcripts were reviewed again using the refined coding framework. Coding consistency reached 92%, and unresolved differences were discussed and checked against the original transcripts until consensus was reached. NVivo 12 Plus was used to facilitate data organization, retrieval, and management.

## Results

### Common method variance

Potential common method bias was examined with Harman’s single-factor test. The largest unrotated factor explained 21.86% of the total variance, below the conventional 40% threshold suggested in prior work (Podsakoff et al., 2003).

### Descriptive statistics and correlation analysis

Descriptive statistics, score ranges, and zero-order correlations among the focal study variables are reported in Table 1. Descriptive information for the demographic covariates is provided in Supplementary Table S1. Among the focal study variables, children’s emotional competence was positively correlated with mindful parenting, parental involvement, grandparent support, marital satisfaction, and SES, and negatively correlated with parental depression, parental phubbing, and child social withdrawal. Mindful parenting and parental involvement were positively intercorrelated; both were positively associated with the family resource variables and negatively associated with parent- and child-risk variables. Full coefficients are provided in Table 1 and are therefore not repeated in the text.

**Table 1:** Descriptive statistics and zero-order correlations among the main study variables (*n* = 4,020)

Variable	M	SD	Min-Max	1	2	3	4	5	6	7	8	9
1. Emotional competence	3.642	0.555	1.000–5.000	-								
2. Parental depression	1.573	0.462	1.000-3.900	− 0.169^***^	-							
3. Parental phubbing	2.713	0.927	1.000–7.000	− 0.058^***^	0.461^***^	-						
4. SES	0.000	1.000	-3.386-1.576	0.121^***^	− 0.197^***^	0.002	-					
5. Grandparent support	4.946	1.291	1.000–7.000	0.252^***^	− 0.276^***^	− 0.124^***^	0.149^***^	-				
6. Marital satisfaction	3.725	0.711	1.000–5.000	0.175^***^	− 0.457^***^	− 0.226^***^	0.235^***^	0.395^***^	-			
7. Child social withdrawal	2.011	0.641	1.000-4.800	− 0.152^***^	0.343^***^	0.290^***^	− 0.054^***^	− 0.133^***^	− 0.280^***^	-		
8. Mindful parenting	2.883	0.518	1.000–4.000	0.347^***^	− 0.275^***^	− 0.091^***^	0.237^***^	0.458^***^	0.342^***^	− 0.189^***^	-	
9. Parental involvement	2.642	0.653	1.000–4.000	0.316^***^	− 0.190^***^	− 0.077^***^	0.245^***^	0.352^***^	0.329^***^	− 0.114^***^	0.455^***^	-

*SES* family socioeconomic status. Demographic characteristics and coding of the covariates are reported in Supplementary Table S1. Only the lower triangle of the correlation matrix is presented. ^*^p < .05, ^**^p < .01, ^***^p < .001

### Hierarchical regression results

Hierarchical regression was used to identify factors related to children’s emotional competence while controlling for covariates. Variables were entered in six blocks: parent and child demographic variables, parental characteristics, family resources, child characteristics, mindful parenting, and parental involvement. The collinearity diagnostics did not indicate multicollinearity concerns, as all VIF values were below 10 and the maximum VIF was 1.637 (James et al., 2013).

All six regression models were statistically significant, and each successive block accounted for a significant increment in explained variance (Table 2). When first entered into the models, parental depression and child social withdrawal were negatively associated with children’s emotional competence, whereas SES, grandparent support, marital satisfaction, mindful parenting, and parental involvement were positively associated; parental phubbing was not statistically significant.

**Table 2:** Factors associated with children’s emotional competence (*n* = 4,020)

Variables	Model 1	Model 2	Model 3	Model 4	Model 5	Model 6
Demographic Covariates						
Parent age	0.032^*^	0.022	0.028	0.028	0.036^*^	0.029
Parent gender	-0.054^***^	-0.054^***^	-0.068^***^	-0.061^***^	-0.042^**^	-0.041^**^
Child age	0.128^***^	0.128^***^	0.133^***^	0.126^***^	0.125^***^	0.132^***^
Child gender	-0.052^***^	-0.053^***^	-0.056^***^	-0.053^***^	-0.051^***^	-0.054^***^
Parental Characteristics						
Parental Depression		-0.177^***^	-0.079^***^	-0.063^***^	-0.040^*^	-0.047^*^
Parental Phubbing		0.022	0.015	0.028	0.017	0.019
Family Resources						
SES			0.067^***^	0.068^***^	0.030^*^	0.009
Grandparent support			0.205^***^	0.205^***^	0.109^***^	0.084^***^
Marital satisfaction			0.055^**^	0.042^*^	0.010	-0.019
Child Characteristics						
Child social withdrawal				-0.079^***^	-0.059^***^	-0.059^***^
Mindful parenting					0.265^***^	0.204^***^
Parental involvement						0.189^***^
*R* ^2^	0.024	0.053	0.107	0.113	0.163	0.189
Adjusted *R*^2^	0.023	0.051	0.105	0.110	0.161	0.187
∆*R*^2^	0.024	0.028	0.055	0.005	0.051	0.026
*F* (*p*)	24.991^***^	37.072^***^	53.640^***^	50.848^***^	70.989^***^	77.833^***^
∆*F* (*p*)	24.991^***^	59.771^***^	82.272^***^	23.063^***^	241.846^***^	128.311^***^

Values are standardized regression coefficients (*β*). Parent gender and child gender were coded as 0= female and 1 = male. ^*^p < .05, ^**^p < .01, ^***^p < .001

In the final model, child age, grandparent support, mindful parenting, and parental involvement were positively associated with children’s emotional competence, whereas parent gender, child gender, parental depression, and child social withdrawal were negatively associated. Parent age, parental phubbing, SES, and marital satisfaction were not statistically significant in the final model. The final model explained 18.9% of the variance in children’s emotional competence. Full model coefficients and significance tests are reported in Table 2.

### Network analysis results

Prior to estimating the network, each network variable was residualized for parent age, parent gender, child age, and child gender; these covariates were therefore excluded from the node set. A Gaussian graphical model was estimated with EBICglasso in qgraph, using γ = 0.50 as the tuning parameter and applying threshold = TRUE. The network included 18 nodes organized into five pre-defined communities: Parental Characteristics (PD, PP1, PP2, PP3, PP4), Family Resources (GS, MS, SES), Child Characteristics (SW, EC1, EC2, EC3, EC4), Mindful Parenting (MP1, MP2), and Parental Involvement (PI1, PI2, PI3).

**Fig. 1:**
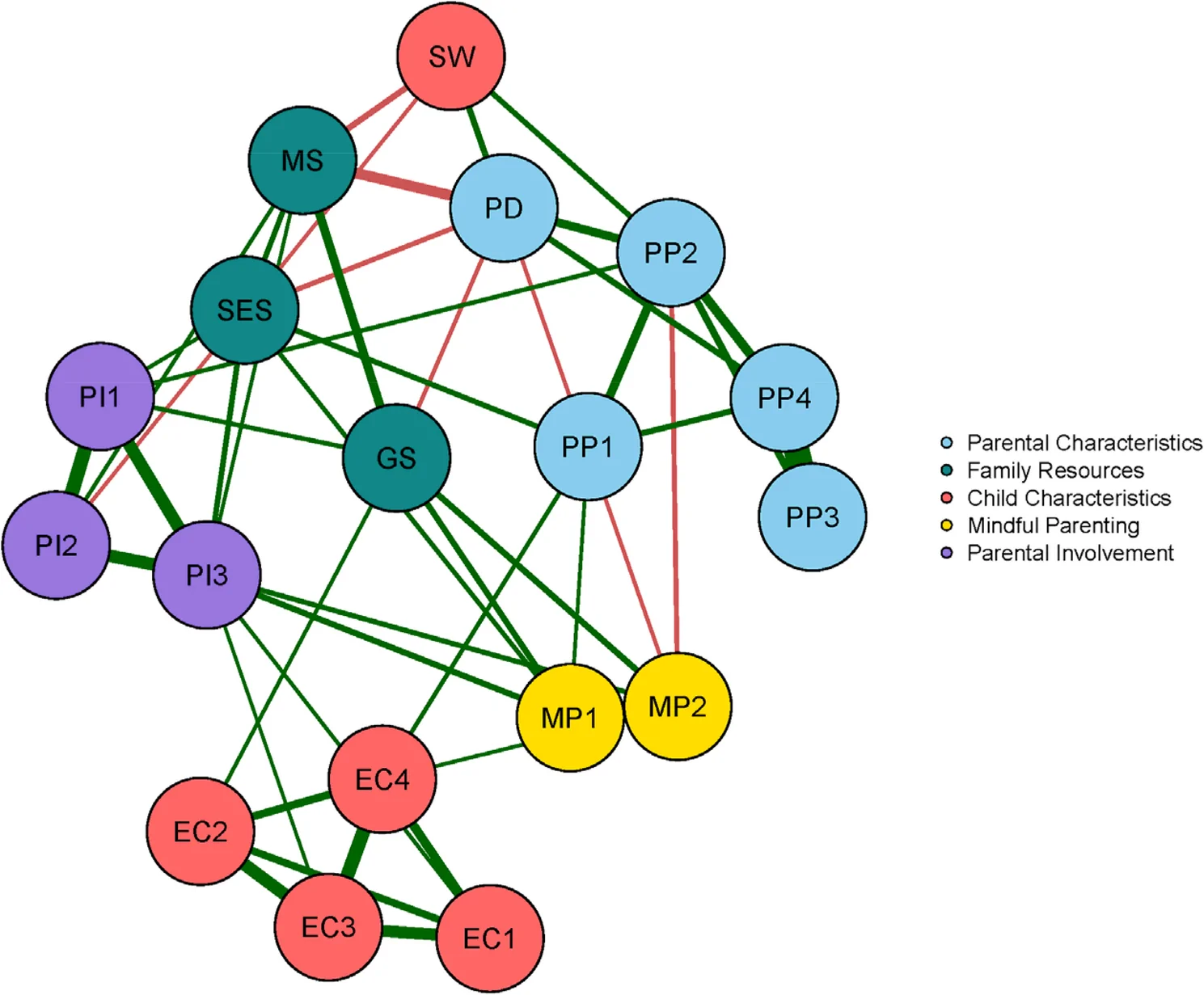
Estimated network of parent characteristics, family resources, child characteristics, and parenting processes. Note. PD, parental depression; PP1, phone separation anxiety; PP2, interpersonal conflict; PP3, self-isolation; PP4, problem recognition; GS, grandparent support; MS, marital satisfaction; SES, socioeconomic status; SW, child social withdrawal; EC1, emotional understanding; EC2, emotional regulation; EC3, emotional expression; EC4, emotional awareness; MP1, mindful discipline; MP2, present-moment attention; PI1, home-kindergarten communication; PI2, kindergarten-based involvement; PI3, home-based involvement. PA, parent age; PG, parent gender; CA, child age; and CG, child gender, were controlled as covariates and are not displayed as network nodes. Green edges indicate positive regularized partial correlations, whereas red edges indicate negative regularized partial correlations; thicker edges denote larger absolute edge weights

Figure 1 presents the estimated GGM (EBICglasso; γ = 0.50). Each edge represents a regularized partial correlation after conditioning on all remaining variables. The network contained 47 nonzero edges, with a density of 0.307. At the level of point estimates, larger associations were concentrated mainly within communities. Pairwise bootstrap difference tests indicated that the PP3-PP4 edge was significantly larger than the MP1-MP2 edge, which was in turn significantly larger than the PI1-PI2 edge. Cross-community connections were observed primarily between mindful parenting and parental involvement and between these parenting dimensions and the Family Resources community. Full edge weights are reported in Supplementary Table S3, and pairwise edge-weight difference tests are presented in Supplementary Fig. S4.

**Fig. 2:**
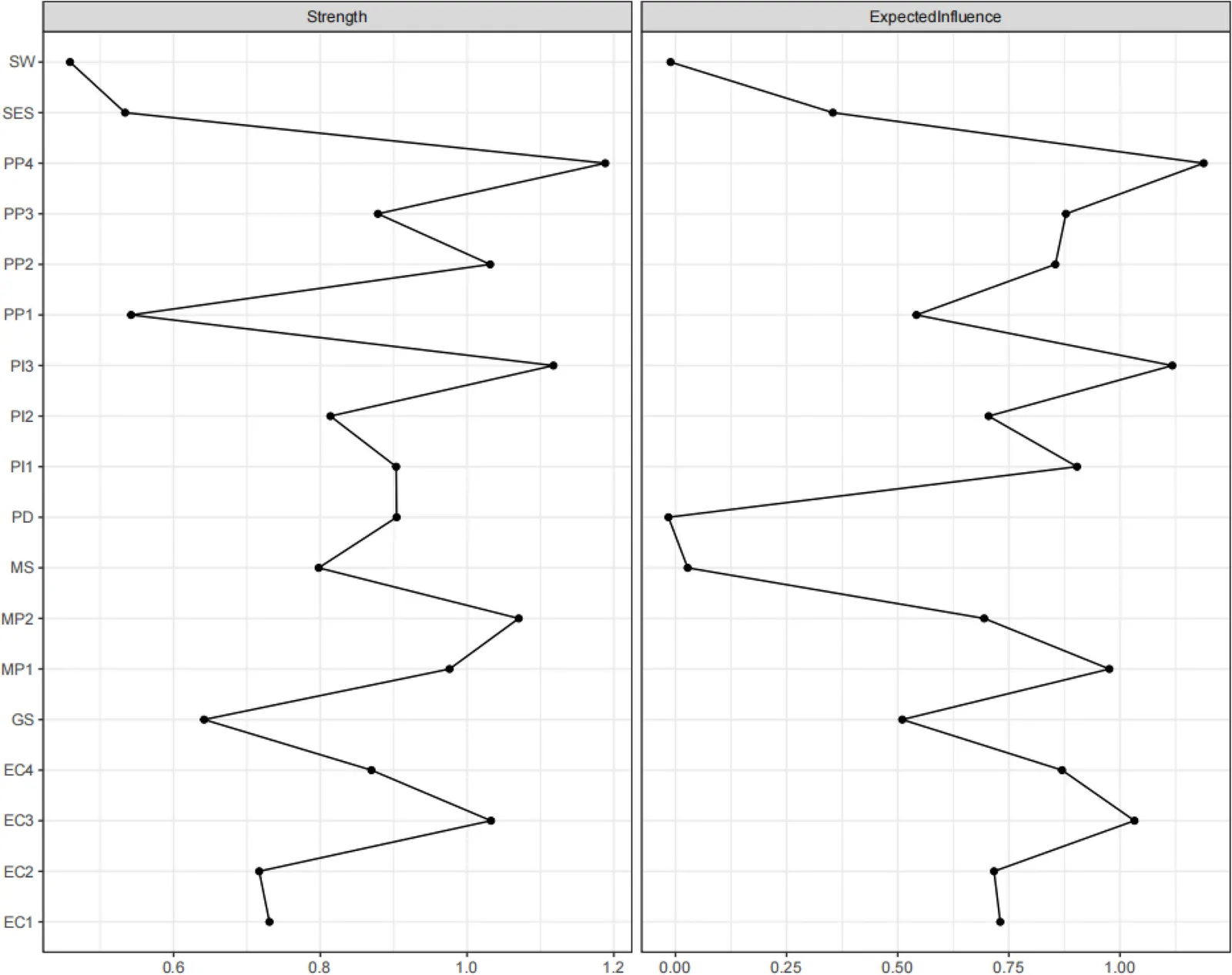
Centrality indices of the estimated network. Note. The left panel presents node strength, and the right panel presents expected influence

Figure 2 summarizes node strength and expected influence. PP4 had the largest point estimate of strength, but it was not significantly different from PI3; PP4 was significantly higher than MP2, whereas PI3 and MP2 did not differ significantly. For expected influence, PP4 had the largest point estimate but did not differ significantly from PI3. PP4 was significantly higher than EC3, whereas PI3 did not differ significantly from EC3 or MP1. These findings indicate that several higher-ranked nodes were not statistically distinguishable and should therefore not be interpreted as uniquely dominant. Strength and expected influence both showed good stability (CS = 0.750; Supplementary Fig. S2). Complete difference-test results are presented in Supplementary Figs. S5 and S6.

**Fig. 3:**
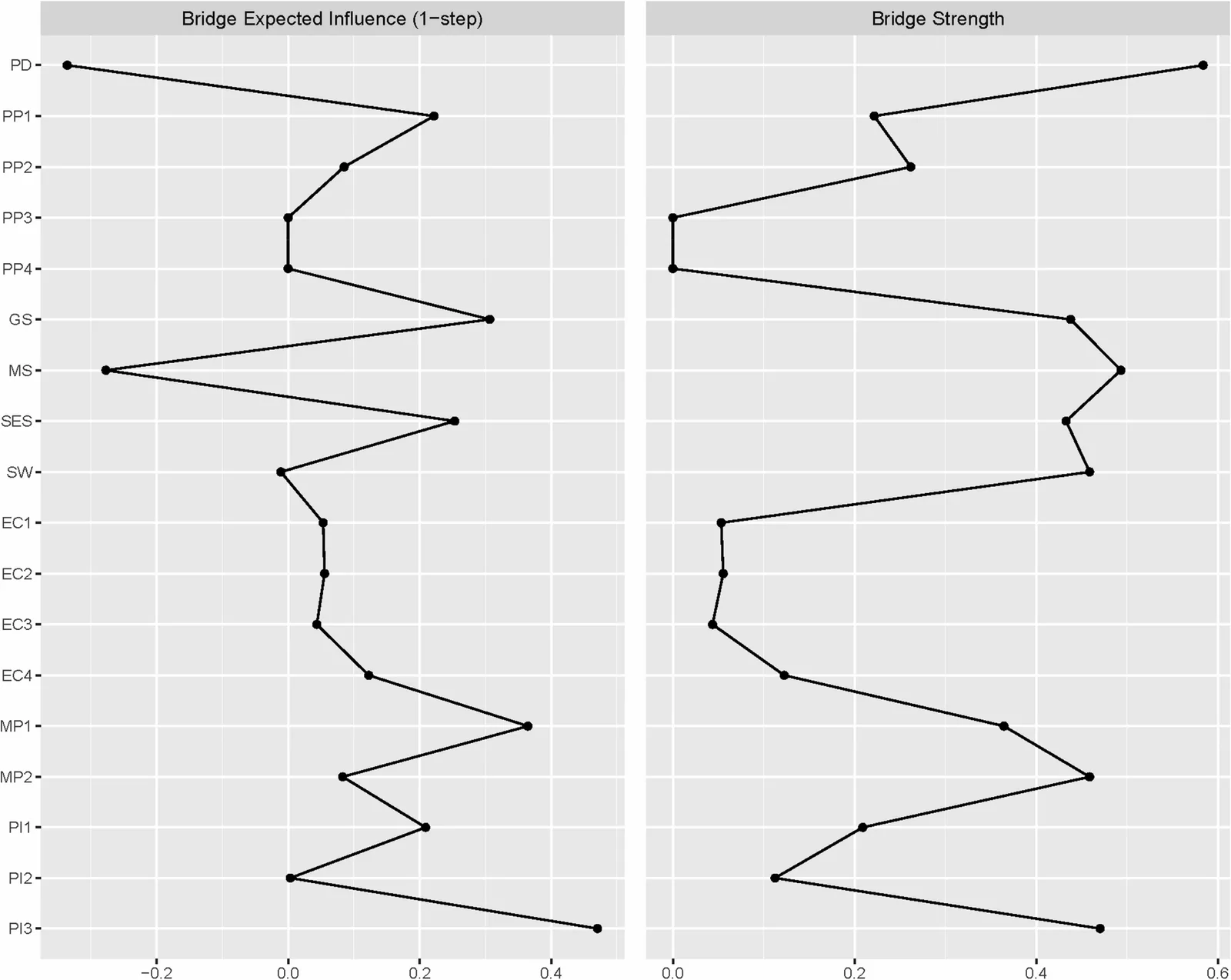
Bridge centrality indices of the estimated network. Note. The left panel presents one-step bridge expected influence, and the right panel presents bridge strength

Figure 3 presents one-step bridge expected influence and bridge strength. PI3, MP1, and GS had the largest positive bridge expected-influence point estimates, but their estimates did not differ significantly from one another. These nodes were therefore interpreted as occupying relatively prominent, rather than uniquely dominant, positive cross-community positions. PD and MS showed negative bridge expected influence. Bridge expected influence demonstrated good stability (CS = 0.672; Supplementary Fig. S1), and its pairwise difference-test results are presented in Supplementary Fig. S7.

Network robustness was assessed using case-dropping and nonparametric bootstrap procedures, each with 1,000 resamples. Edge-weight accuracy is presented in Supplementary Fig. S3; additional centrality-stability and pairwise difference-test results are provided in Supplementary Figs. S1, S2, and S4-S7. Taken together, the network results indicated that conditional associations were concentrated mainly within communities, with additional cross-community connections involving mindful parenting, parental involvement, and the Family Resources community. In addition, the exploratory Network Comparison Test indicated no significant respondent-gender differences in overall network structure (*M* = 0.125, *p* = .880) or global strength (*S* = 1.355, *p* = .256). No individual edge differences remained significant after Benjamini-Hochberg correction, and no significant respondent-gender differences were found in node strength, expected influence, or bridge expected influence.

### Constructivist grounded theory results

**Fig. 4:**
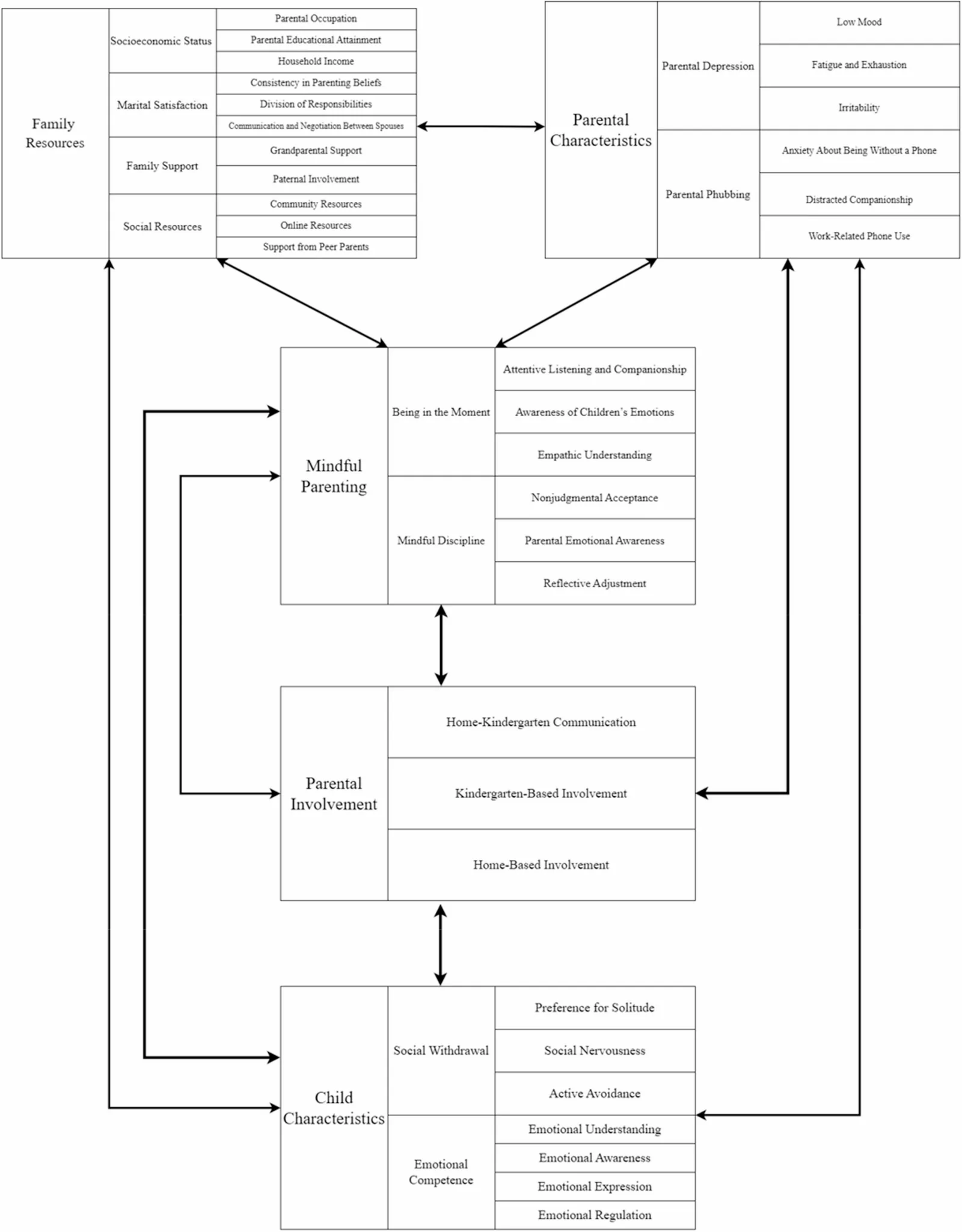
Interpretive relational model of caregiving in parent-grandparent co-parenting families. Note. The arrows represent relationships described and interpreted in parents’ accounts; they do not indicate tested causal, mediating, reciprocal, or temporal pathways.

Based on the parent interview data, the constructivist grounded-theory analysis generated an interpretive relational model of family caregiving within parent-grandparent co-parenting families (Fig. 4). Initial coding generated 67 codes, which were consolidated through focused coding into 32 focused codes and organized into five overarching categories: family resources, parent characteristics, mindful parenting, parental involvement, and child characteristics. Rather than simply reproducing Belsky’s broad categories, relational coding elaborated how parents connected these categories in everyday caregiving. The resulting model highlighted intergenerational negotiation, the dual supportive and potentially conflicting roles of grandparental involvement, digital distraction in caregiving, the complementarity of mindful parenting and parental involvement, home-kindergarten communication, and parents’ adjustments in response to child behavior. The model should therefore be understood as a substantive qualitative account constructed from participants’ narratives, rather than as an empirical confirmation of Belsky’s model or a tested causal mechanism.

#### Family resources

The category of family resources included four focused dimensions: SES, family support, marital satisfaction, and social resources. SES consisted of parents’ educational attainment, occupation, and household income. Parents’ occupational backgrounds were mainly reflected in differences in the amount of time available for companionship. One parent stated, *“By the time we get home from work*,* it is almost eight o’clock*,* so we basically have only about one hour to spend with the child”* (T06). Parents’ educational levels and occupational experiences also influenced how they responded to children’s emotions and developmental needs. A parent working in an education-related field noted, *“My child used to stay awake past ten o’clock at night*,* crying and making a fuss. I knew that losing my temper would not work*,* so I made a bedtime agreement with her and stopped repeatedly urging her verbally. Gradually*,* her bedtime emotions and cooperation improved”* (T20).

Marital satisfaction was reflected in consistency in parenting beliefs, division of responsibilities, and communication and negotiation between spouses. Consistency in parenting beliefs referred to the extent to which both spouses maintained shared basic parenting principles. One parent stated, *“Both my child’s father and I have clear principles in mind. For example*,* when one of us is educating the child*,* the other will not interrupt easily”* (T02). Division of responsibilities referred to both spouses’ joint investment in companionship and caregiving. One parent used the metaphor of planting a tree: *“The soil is our marital relationship. We rarely quarrel*,* and we provide the child with a warm family environment. The sunlight and rain are the time and energy we devote to companionship*,* as well as scientific educational approaches”* (T20). Communication and negotiation between spouses referred to discussing disagreements privately and adjusting parenting approaches afterward. One parent said, *“Even if we have different opinions*,* we do not argue in front of the child. Afterward*,* we communicate privately and adjust our educational approach together”* (T02).

Family support was most visible in parent-grandparent coordination and paternal involvement. Grandparent support could reduce parents’ caregiving pressure and provide children with additional emotional soothing, but it could also create coordination challenges when generations differed in discipline rules or interpretations of children’s distress. One parent said, *“We usually cooperate in discipline by playing ‘good cop and bad cop.’ For example*,* when the child loses her temper*,* I insist on the rule*,* while her grandmother holds her and helps her calm down*,* and then we work together to think of a solution”* (T19). However, another parent reported, *“From age one to three*,* the child was mainly cared for by his grandparents on both sides. They spoiled him and did not set rules for him*,* which made him rather indulged and prone to tantrums”* (T18). These accounts illustrated variation in how parents perceived grandparental support, particularly in relation to the consistency of caregiving expectations, discipline practices, and emotional responses across generations. Paternal involvement was also reflected in fathers’ engagement in companionship, soothing, and rule modeling. One parent noted, *“Sometimes the child’s father would say threatening things like*,* ‘If you do this again*,* I will hit you.’ At that moment*,* the child would become afraid to speak or express himself”* (T12).

Social resources included community resources, online resources, and support from peer parents. Community resources referred to family education courses, libraries, and public welfare activities. One parent mentioned that they would *“make use of social resources for parenting*,* such as family education courses offered by the Women’s Federation and libraries*,* which teach us how to communicate and how to effectively and quickly ease crying”* (T06). Online resources referred to parents’ use of the internet to obtain parenting information. One parent reflected, *“Sometimes I really do not know how to guide him to express his emotions*,* so I go online to see what teachers and professors say”* (T12). Support from peer parents was reflected in exchanges of parenting experiences among parents. One parent said, *“Sometimes I ask Xiaoyu’s mother how she handles it. She is quite experienced”* (T19).

#### Parental characteristics

Parent characteristics included two focused dimensions: depressive symptoms and phubbing. Depressive symptoms were mainly manifested as low mood, fatigue and exhaustion, and irritability. Low mood was reflected in parents’ feelings of helplessness during caregiving. One parent stated, *“Sometimes I am already very tired*,* and when the child throws a tantrum and refuses to talk*,* I do not have the energy to communicate with him slowly. I also need time to calm down”* (T17). Fatigue and exhaustion mainly stemmed from work pressure, limited time, and the sustained demands of caregiving. One parent said, *“There are times when my energy is very low. I may scold them or even hit them*,* but I know this definitely has a negative impact on children’s emotional expression”* (T06). Irritability was reflected in parents’ greater tendency to lose their temper when children cried, delayed, or came into conflict with rules. One parent noted, *“I have lost my temper in those situations before. Afterward*,* I felt that I should not have done that*,* so I apologized to the child and said that Mom had failed to control her emotions. We then discussed how to make this happen less often in the future”* (T12).

Phubbing was mainly manifested as anxiety about being without a phone, distracted companionship, and work-related phone use. Anxiety about being without a phone reflected parents’ difficulty disengaging from their phones because of work, social communication, or habit. One parent said, *“We cannot be without our phones because we are afraid of missing messages. When I am busy*,* I let him play with toys by himself*,* and sometimes I may overlook his emotions”* (T18). Distracted companionship referred to situations in which parents were physically present but their attention was not directed toward the child. One mother said, *“Most of the time*,* the child’s father plays on his phone by himself. The child does not take the initiative to play with him*,* and even when the child cries and calls for him*,* the father does not really respond”* (T11). Work-related phone use referred to parents’ need to handle work messages while caring for the child. One parent said, *“When I am spending time with him*,* I try to put my phone down. If I need to use it for work*,* I explain it clearly to him and tell him that Mom is working”* (T12).

#### Mindful parenting

Mindful parenting included two focused dimensions: being in the moment and mindful discipline. Being in the moment included attentive listening and companionship, awareness of children’s emotions, and empathic understanding. Attentive listening and companionship were reflected in parents’ ability to maintain attention during interactions and seriously accept children’s expressions. One parent stated, *“I try to listen more to what the child says and not deny him. Even if what he says at the moment is wrong*,* I will still communicate with him slowly and try to understand”* (T01). Awareness of children’s emotions referred to parents’ observation of changes in children’s emotions and their responsive actions. One parent mentioned that when the child became emotionally intense, they would first take the child to a room to calm down and, after the child had settled, *“listen to him talk about his thoughts and communicate with him”* (T09). Empathic understanding was reflected in parents’ attempts to understand the needs underlying children’s behaviors. One parent said, *“We do not focus only on the behavior itself*,* but try to understand the real need behind it and then help him find an appropriate solution”* (T02).

Mindful discipline included nonjudgmental acceptance, parental emotional awareness, and reflective adjustment. Nonjudgmental acceptance referred to parents first acknowledging children’s emotions before providing guidance. One parent said, *“If the situation is understandable*,* I first accept his emotions and tell him that everyone has bad moods and that this is normal. Then I guide him to draw what makes him unhappy and throw the bad emotions away”* (T10). Parental emotional awareness was reflected in parents’ awareness and expression of their own emotions during discipline. One mother noted, *“I will directly tell him*,* ‘Mom is very tired now’ or ‘Mom is very angry now*,*’ and explain why”* (T01). Reflective adjustment referred to parents’ modification of their own responses based on parent-child interactions. One parent mentioned that after losing their temper, they would apologize to the child, explain that *“Mom failed to control her emotions*,*”* and discuss with the child how to reduce such situations in the future (T12).

#### Parental involvement

Parental involvement included three focused dimensions: home-kindergarten communication, kindergarten-based involvement, and home-based involvement. Within these parent-reported accounts, teachers appeared primarily as communication partners who provided information about children’s experiences and emotional behavior in kindergarten. Teacher feedback was interpreted as part of parents’ home-kindergarten communication and subsequent support at home; it was not treated as evidence of an independent teacher effect or a teacher-mediated pathway between parents and children. Home-kindergarten communication was reflected in parents’ understanding of children’s kindergarten experiences through teacher feedback. One parent said, *“The teacher gives feedback on the child’s state at school and discusses how to guide the child to learn to express emotions”* (T12). Kindergarten-based involvement included open days, parent-child activities, class activities, and parent teaching-assistant activities. One parent noted, *“During the open day*,* the teacher shared that developing children’s confidence and communication requires patient guidance again and again”* (T02). Home-based involvement was reflected in parents’ companionship and support when children dealt with frustration and rule-related issues in the family context. One parent stated, *“What parents can do is provide enough security*,* letting him know that even if he makes mistakes or fails*,* Mom and Dad will accompany him in improving. Later*,* when he encountered problems*,* he became less anxious”* (T14).

#### Child characteristics

Child characteristics included two focused dimensions: social withdrawal and emotional competence. Social withdrawal included a preference for solitude, social nervousness, and active avoidance. Preference for solitude was reflected in children’s tendency to engage in activities alone. One parent noted, *“He does not take much initiative to communicate with other children and says that he likes playing by himself”* (T14). Social nervousness was reflected, in some parents’ accounts, in individual children’s reluctance to express their needs when interacting with teachers, unfamiliar adults, or in group situations. One mother recalled, *“Once*,* his finger was caught by a chair in the classroom*,* but he did not dare tell the teacher. I only found out when I picked him up that evening*,* and his finger was already bruised”* (T09). This pattern was interpreted as a context-specific manifestation of social nervousness rather than as evidence of a local cultural norm or a deviation from such a norm. Active avoidance was reflected in children’s withdrawal and nonparticipation in group activities or unfamiliar situations. One parent described, *“When he first entered the junior class*,* he had severe separation anxiety. For the first month*,* he sat at the back crying and did not participate in activities”* (T20).

Emotional competence included emotion understanding, emotional expression, emotional awareness, and emotion regulation. Emotion understanding was reflected in children’s ability to explain the causes of their own or others’ emotions: *“She can say why she is angry*,* for example*,* because she feels that others did not listen to her*,* or because something that belonged to her was taken away*,* so she feels unhappy”* (T15). Emotional expression referred to children’s ability to use language to express their emotions, needs, and experiences. One parent said, *“He can now clearly express the reason for and process of becoming angry”* (T17). Emotional awareness was reflected in children’s ability to identify their own emotional states: *“She will run to the room by herself and ask all of us to leave*,* saying that she needs to calm down”* (T15). Emotion regulation referred to children’s ability to gradually recover from crying, anger, frustration, or anxiety. One parent said, *“His emotion management is much better than before*,* and he rarely loses his temper now”* (T17).

#### Relationships among categories: relational coding and interpretive propositions

Relational coding identified how parents connected the five overarching categories in their accounts. Family resources were frequently discussed alongside parents’ stress, emotional availability, and everyday caregiving conditions. Parents also connected access to family support and social resources with how they obtained, interpreted, and applied parenting guidance. Depression, fatigue, and phubbing were described in relation to difficulties sustaining attentive companionship and emotionally responsive interaction, whereas mindful parenting accounts emphasized emotional awareness, acceptance, and reflective adjustment. Mindful parenting and parental involvement were described as connected but distinguishable aspects of caregiving: the former concerned the emotional and regulatory quality of parents’ responses, whereas the latter concerned the settings and activities through which caregiving was enacted. Child social withdrawal and emotional competence were positioned as child-related conditions to which parents adjusted their responses and involvement. These relational patterns formed the interpretive structure of the qualitative model; they do not establish temporal ordering, mediation, reciprocal effects, or causal feedback mechanisms.

#### Divergent and negative cases

To clarify the boundaries of the model, the present study further examined cases that were inconsistent with the predominant relational pattern. The first type of divergence emerged in the link between parent-grandparent coordination and children’s emotional development. Although grandparent support generally helped reduce caregiving pressure, inconsistent interpretations of challenging experiences between parents and grandparents could also affect how children responded to frustration. One parent mentioned that when grandparents occasionally picked up and dropped off the child for basketball lessons, they felt concerned about the intensity of the training and said things such as *“it is hard” and “you must be tired.”* Afterward, the child began to say that they did not want to continue attending the lessons (T01). The second type of divergence appeared in kindergarten-based activity participation. Although some activities were intended to promote parent-child cooperation, children’s actual participation was limited. When discussing a themed kindergarten activity, one parent said, *“It was actually difficult for children to participate in this kind of activity. The original intention was parent-child cooperation and communication*,* but in practice it became a task for parents”* (T03). The third type of divergence reflected contextual differences in children’s emotional competence. Some children were able to follow rules and remain emotionally stable in kindergarten but were more likely to display emotional externalization at home. One parent described, *“When he encounters difficulties in kindergarten*,* based on the teacher’s feedback*,* he does not show obvious emotional fluctuations and will persist in completing the task. But when he encounters difficulties at home*,* such as failing to build with blocks or when things do not go smoothly*,* he first loses his temper and then asks us for help”* (T16).

Overall, these divergent cases indicated that the relationships among family resources, parental regulation, parental involvement, and children’s emotional competence were contingent rather than uniform. They refined the boundaries of the proposed model by showing that the presence of family support or parental involvement was not invariably accompanied by greater emotional competence and that the observed patterns varied according to caregiving consistency, children’s actual participation, and interactional context. These cases are presented as qualitative contrasts that informed model development, rather than as tests of causal effects.

## Discussion

Taken together, the findings provided different levels of support for the five hypotheses. H1 and H2 were supported at the construct level: mindful parenting and parental involvement remained positively associated with children’s emotional competence. At the dimension level, home-based involvement, mindful discipline, and grandparent support showed relatively prominent positive bridge expected-influence point estimates, although these estimates did not differ significantly from one another. H3 was partially supported: SES, grandparent support, and marital satisfaction were positively associated with emotional competence when first entered into the regression model, but only grandparent support remained significant in the final model. H4 was partially supported: parental depression remained negatively associated with emotional competence, whereas parental phubbing did not show an independent association in the regression model. H5 was supported: child social withdrawal remained negatively associated with emotional competence. Across these findings, parents were the sole data providers; children were the focal group whose characteristics and emotional competence were parent-reported, while teachers and grandparents were contextual actors rather than independently assessed participants.

Importantly, neither the cross-sectional quantitative analyses nor the parent interviews tested mediation, moderation, temporal ordering, or causal mechanisms. References in the following discussion to processes, connections, or feedback should therefore be understood as interpretive relationships described in parents’ accounts and as theoretically informed propositions, rather than as empirically established mechanistic pathways.

### Mindful parenting and parental involvement as proximal family processes

The results indicated that mindful parenting and parental involvement were positively related to young children’s emotional competence in the regression models; the network analysis further showed that mindful discipline, home-based involvement, and grandparent support had relatively prominent positive bridge expected-influence point estimates; and the qualitative findings elaborated how these associations were reflected in parents’ everyday caregiving accounts. This finding echoes previous evidence that mindful parenting is associated with reduced reactivity, greater acceptance, and more supportive parent-child interaction patterns (Duncan et al., 2009; McKee et al., 2018). It also aligns with evidence that family involvement and parent-focused involvement programs are associated with children’s social-emotional development across early childhood contexts (Cosso et al., 2022; Fantuzzo et al., 2004; Wang, 2026).

From the perspective of Belsky’s process model of parenting, mindful parenting can be understood as a proximal regulatory process related to how parents notice children’s emotional cues, manage their own reactions, and respond with acceptance and guidance (Belsky, 1984; Taraban & Shaw, 2018). Parental involvement, in turn, represents the concrete contexts in which parents engage with children’s emotional experiences through companionship, communication, and home-kindergarten collaboration. Thus, the two processes appear complementary rather than interchangeable: mindful parenting reflects the emotional quality of parental responses, whereas parental involvement reflects the practical enactment of caregiving across everyday settings.

The Chinese preschool family context further helps explain why these processes were salient. In many Chinese families, young children’s emotional experiences are distributed across home, kindergarten, and multigenerational caregiving arrangements (Chen et al., 2011; Xu et al., 2024). Parents are therefore expected to respond to children’s emotions at home, communicate with teachers, and coordinate caregiving expectations with other family members. Interview accounts illustrated this pattern: parents described accepting children’s emotions before guidance, repairing interactions after losing control, providing security during frustration, and using teacher feedback to understand children’s emotional states. These findings suggest that parent support should not focus only on increasing the frequency of involvement, but should also strengthen the emotional quality of involvement through attentive listening, emotion coaching, reflective discipline, and supportive home-based interaction (England-Mason et al., 2023; Havighurst et al., 2010).

### Parent and child characteristics as relational conditions

Beyond mindful parenting and parental involvement, parent and child characteristics were associated with variation in parent-reported children’s emotional competence. The hierarchical regression showed that child age was consistently positively associated with emotional competence, whereas child gender, child social withdrawal, and parental depression were negatively associated with it. The network analysis further suggested that these variables were not isolated factors, but were embedded in an interconnected system involving family resources, mindful parenting, and parental involvement. The qualitative findings elaborated this pattern. In the interviews, parents described fatigue, low mood, and anxiety alongside reduced patience when responding to children’s emotions, and they described children’s withdrawal or limited emotional expression as making emotional needs more difficult to identify.

This pattern can be understood through the process model of parenting. Parent psychological functioning and child behavioral characteristics are not merely background variables; rather, they shape the conditions under which parent-child interaction takes place (Belsky, 1984; Taraban & Shaw, 2018). Parental depression may reduce emotional availability and responsive sensitivity, whereas child social withdrawal may limit opportunities for emotional expression, communication, and co-regulation (Lovejoy et al., 2000; Rubin et al., 2009). The interview accounts were consistent with the possibility of reciprocal relations: parents described adjusting their responses when children expressed less, while also reporting that sustained stress or emotional exhaustion made supportive responding more difficult (Sameroff & Mackenzie, 2003; Wang & Cheng, 2025). However, neither the temporal ordering nor the reciprocal nature of these relationships was tested. These accounts should therefore be interpreted as qualitative propositions for future investigation rather than evidence of an established feedback mechanism. The findings concerning parental phubbing are particularly important to interpret within this system. Although parental phubbing was significantly correlated with children’s emotional competence and retained connections in the network analysis, its independent association was not significant in the hierarchical regression. This does not necessarily indicate that parental phubbing is unrelated to family functioning. Rather, the nonsignificant regression coefficient indicates that its zero-order association with children’s emotional competence was not independent of the other variables included in the model. Parental phubbing may covary with parental stress, attentional distraction, emotional exhaustion, or lower-quality involvement (McDaniel & Radesky, 2018; Zhang et al., 2024). This issue may be especially complex in Chinese family contexts, where smartphones are often used simultaneously for work communication, home-kindergarten contact, family coordination, and personal relaxation. Parental phubbing should therefore not be equated automatically with parental neglect, because smartphone use may occur alongside work-family boundary demands, parenting pressure, family coordination, and personal use in digitalized everyday life (McDaniel, 2019; van Zoonen et al., 2020). Consistent with this interpretation, the developmental implications of screen use depend not only on its duration but also on its content, context, and nature, as well as on parents’ own media-use patterns (Fitzpatrick et al., 2023).

### Parent-grandparent co-parenting and family resources as contextual conditions

Family resources formed another important set of contextual conditions for children’s emotional competence. The hierarchical regression showed that grandparent support remained positively associated with children’s emotional competence across models, whereas SES and marital satisfaction became nonsignificant after mindful parenting and parental involvement were included. The network analysis also indicated that variables in the Family Resources community were connected with parent characteristics, parenting processes, and children’s emotional competence. The qualitative findings further clarified that the significance of resources lies not in their mere presence, but in how they enter everyday caregiving. Some parents described grandparent support as reducing caregiving pressure and allowing them more energy to accompany their children, whereas others noted that inconsistent rules and different educational beliefs across generations could create additional coordination costs (Li et al., 2020; Xiao, 2016).

These findings suggest that family resources are better understood as contextual correlates rather than direct determinants of children’s emotional competence. SES, marital satisfaction, and grandparent support were positively associated with emotional competence when first entered into the regression model, but only grandparent support remained significant in the final model. The attenuation of the SES and marital-satisfaction coefficients may reflect variance shared with mindful parenting and parental involvement. Because indirect effects were not tested, this pattern should not be interpreted as evidence that family resources operate through these proximal parenting processes.

This interpretation is especially relevant to parent-grandparent co-parenting in the Chinese context. In many Chinese families with preschool-aged children, caregiving is not carried out by parents alone, but is embedded in intergenerational co-caregiving and home-kindergarten collaboration (Chen et al., 2011; Sun et al., 2025). Grandparent support may reduce parents’ caregiving pressure and create more emotional space for mindful responses and sustained involvement, which is consistent with evidence linking grandparents’ involvement to lower maternal parenting stress and stronger family functioning in Chinese three-generation families (Wu et al., 2025). At the same time, parent-grandparent co-parenting may also require parents to coordinate rules, discipline practices, and emotional responses across generations. Parents’ accounts suggested that perceptions of grandparent support varied according to the amount of help provided and the extent to which caregiving expectations, discipline practices, and emotional responses were coordinated across generations. Accordingly, family support services should not focus only on individual parenting skills. They should also help families reduce parental caregiving pressure, promote spousal and intergenerational coordination, minimize conflicts in educational beliefs, and transform available resources into a more sensitive and consistent emotional response environment.

### Theoretical and practical implications

Theoretically, the qualitative analysis offers a context-specific substantive elaboration of Belsky’s process model rather than a confirmation of that model. It specifies how parents in Chinese parent-grandparent co-parenting families connected contextual resources, intergenerational coordination, parental psychological functioning, mindful parenting, parental involvement, and child characteristics in their accounts of everyday caregiving. It also introduces contextual elements that are not fully specified in the original model, including digitalized caregiving, home-kindergarten communication, intergenerational negotiation, and divergent cases in which family support or involvement did not correspond to the expected child outcome. These relationships constitute theoretically informed propositions generated from the qualitative data and require testing through longitudinal and mechanism-focused research.

Practically, the findings suggest that efforts to promote preschool children’s emotional competence should not focus only on increasing the amount of parental involvement, but also on improving the emotional quality of that involvement. Parenting programs and family education services may benefit from emphasizing mindful listening, emotion recognition, nonjudgmental acceptance, reflective discipline, and high-quality companionship. At the same time, future parenting programs and family-support initiatives may consider the broader family system by addressing caregiving stress, supporting spousal and intergenerational coordination, facilitating home-kindergarten communication, and helping parents sustain responsive attention to children’s emotional cues in digitalized daily life. For Chinese preschool families, such system-oriented support may be more meaningful than recommendations directed only at individual parenting behaviors.

### Limitations and future directions

Several limitations should be considered. First, the cross-sectional survey cannot establish causal direction, temporal ordering, mediation, or developmental change. The network analysis identified conditional associations rather than causal pathways, and the qualitative model was based on parents’ retrospective accounts. Its relational directions should therefore be interpreted as theoretically informed propositions rather than verified sequential or reciprocal mechanisms. Longitudinal and multiwave mixed-methods research is needed to examine these temporal relationships more directly.

Second, most variables were reported by the same parent. In particular, the MIPQ captures parents’ perceptions of how frequently they demonstrate mindful discipline and present-moment attention, rather than directly observed parenting behavior or children’s experiences of these practices. Its two-factor structure also represents a focused operationalization of mindful parenting and may not fully capture related dimensions such as emotion coaching, warmth, or culturally specific forms of responsiveness. The findings should therefore be interpreted as associations involving parent-reported mindful parenting. Future studies could combine the MIPQ with observational measures, partner reports, ecological momentary assessment, and independent assessments of children’s emotional competence.

Third, although the sample included 40 kindergartens across eight provinces, it was not nationally representative. Children, teachers, and grandparents were also not independently assessed; references to teacher feedback, grandparental caregiving, and children’s behavior reflected parents’ perspectives. Accordingly, individual examples of children’s social nervousness should be understood as context-specific accounts rather than evidence of a broader cultural norm. The respondent-gender network comparison should likewise be viewed as exploratory because of the unequal group sizes. Future research could use more representative sampling and incorporate reports from children, teachers, grandparents, mothers, and fathers.

## Conclusions

This explanatory sequential mixed-methods study examined how mindful parenting and parental involvement were situated within the broader caregiving ecology of Chinese parent-grandparent co-parenting families. At the construct level, mindful parenting, parental involvement, and grandparent support were positively associated with parent-reported preschoolers’ emotional competence in the final regression model, whereas parental depression and child social withdrawal were negatively associated with it. At the dimension level, home-based involvement, mindful discipline, and grandparent support occupied relatively prominent positive cross-community positions, although none emerged as a uniquely dominant bridge. The qualitative findings further illustrated how parents connected emotionally responsive parenting, everyday involvement, intergenerational coordination, parental psychological functioning, and adjustments to children’s behavior in daily caregiving. Taken together, the findings describe an associative family ecology in which preschoolers’ emotional competence is related to both the quality of proximal parenting and the coordination of caregiving resources across generations. Because the evidence was cross-sectional and largely based on parent reports, these relationships should not be interpreted as causal or temporal pathways.

## Supplementary Information

Supplementary Material 1.

## Data Availability

The datasets generated and/or analyzed during the current study are not publicly available because they contain potentially sensitive information provided by caregivers and parent-reported information about preschool children, and public data sharing was not covered by the participants’ informed consent or the institutional ethics approval. De-identified quantitative data may be made available by the corresponding author upon reasonable request, subject to additional ethics approval and the execution of a data-use agreement. The qualitative interview transcripts are not available because complete de-identification cannot fully eliminate the risk of participant identification.
