# Supplementary material for: Mindful parenting, parental involvement, and preschoolers’ emotional competence in parent-grandparent co-parenting families in China: an explanatory sequential mixed-methods study
Source: Psicol Reflex Crit. 2026 Aug 18;39:36. doi: 10.1186/s41155-026-00409-x (PMC13759920; doi:10.1186/s41155-026-00409-x)
Supplement: Supplementary file 1 [file 41155_2026_409_MOESM1_ESM.docx]

Appendix A

Appendix A.1. Family Socioeconomic Status (SES)

Family socioeconomic status was measured using indicators of parental occupation, educational attainment, and household income (Ren, 2010). Parental occupation was coded with reference to the research by Shi and Shen (2007), assigning scores from 1 to 5, ranging from “temporary workers, unemployed individuals, non-skilled workers, and agricultural laborers” to “senior managers, senior professionals, and professional executives.”

Parental educational attainment was categorized into five levels, drawing on the method of Li et al. (2020), and assigned scores from 1 to 5: “middle school or below,” “high school or vocational school,” “associate degree,” “bachelor’s degree,” and “master’s degree or above.” Monthly household income per capita was divided into seven brackets: “≤2000 RMB,” “2001-3000 RMB,” “3001-4000 RMB,” “4001-5000 RMB,” “5001-10,000 RMB,” “10,001-20,000 RMB,” and “>20,000 RMB,” scored from 1 to 7 respectively.

The three indicators were standardized and entered into factor analysis. The analysis yielded one principal factor with an eigenvalue greater than 1, which accounted for 65.70% of the total variance. The factor loadings for parental education, parental occupation, and household income were 0.817, 0.852, and 0.761, respectively. The SES score was calculated as follows: Family SES = (0.817 × Zeducation + 0.852 × Zoccupation + 0.761 × Zincome) / 1.971. The resulting score was then standardized, with higher scores indicating higher SES.

Appendix B

**Table S1.** Demographic characteristics of the quantitative sample (*n* = 4,020)

| **Variable** | **Descriptive statistic** |
| --- | --- |
| **Continuous characteristics** |  |
| Parent age, years | 35.23 (4.60), 21-55 |
| Child age, months | 53.62 (12.63), 24-83 |
| **Questionnaire respondent** |  |
| Mother/female respondent | 3,237 (80.52%) |
| Father/male respondent | 783 (19.48%) |
| **Child class level** |  |
| Lower | 1,259 (31.32%) |
| Middle | 1,304 (32.44%) |
| Upper | 1,306 (32.49%) |
| Other | 151 (3.76%) |
| **Child gender** |  |
| Male | 2,136 (53.13%) |
| Female | 1,884 (46.87%) |
| **Only child** |  |
| Yes | 1,783 (44.35%) |
| No | 2,237 (55.65%) |
| **Family income, RMB per month per capita** |  |
| ≤ 2,000 | 71 (1.77%) |
| 2,001 - 3,000 | 109 (2.71%) |
| 3,001 - 4,000 | 210 (5.22%) |
| 4,001 - 5,000 | 412 (10.25%) |
| 5,001 - 10,000 | 1,276 (31.74%) |
| 10,001 - 20,000 | 1,222 (30.40%) |
| > 20,000 | 720 (17.91%) |
| **Occupation** |  |
| Temporary, unskilled, agricultural, or unemployed | 169 (4.20%) |
| Manual worker, technician, or self-employed | 570 (14.18%) |
| General, service, or administrative staff | 780 (19.40%) |
| Middle-level professional or manager | 1,569 (39.03%) |
| Senior professional or manager | 932 (23.18%) |
| Educational level |  |
| Middle school or below | 123 (3.06%) |
| High school or vocational school | 473 (11.77%) |
| Associate degree | 690 (17.16%) |
| Bachelor’s degree | 2,018 (50.20%) |
| Master’s degree or above | 716 (17.81%) |

**Note.** Continuous variables are presented as *M* (*SD*), range; categorical variables are presented as *n* (%). Parent gender and child gender were coded as 0 = female and 1 = male in the quantitative analyses. Family income refers to monthly household income per capita in RMB. Occupational status reflects the highest occupational level between the mother and father, and educational level reflects the highest educational attainment between the mother and father. SES was constructed from parental education, occupational status, and household income, with higher factor scores indicating higher SES. Percentages may not sum to 100 because of rounding.

**Table S2.** Characteristics of interview participants (*n* = 20)

| **ID** | **Respondent gender** | **Parent age** **(years)** | **Educational level** | **Occupation** | **Monthly income** **(RMB)** | **Child age** **(years)** | **Family structure** | **Interview duration** **(min)** |
| --- | --- | --- | --- | --- | --- | --- | --- | --- |
| T01 | Female | 34 | Bachelor’s degree | Primary school teacher | 5,001-10,000 | 5 | Stem family | 42 |
| T02 | Female | 31 | Associate degree | Unemployed | 2,001-3,000 | 4 | Stem family | 38 |
| T03 | Male | 36 | High school | Self-employed | 4,001-5,000 | 6 | Nuclear family | 41 |
| T04 | Female | 33 | Master’s degree | Secondary school teacher | 10,001-20,000 | 4 | Stem family | 45 |
| T05 | Female | 29 | High school | Temporary worker | 2,001-3,000 | 3 | Stem family | 37 |
| T06 | Female | 35 | Bachelor’s degree | Kindergarten teacher | 5,001-10,000 | 5 | Stem family | 44 |
| T07 | Female | 38 | Associate degree | Self-employed | 4,001-5,000 | 6 | Stem family | 40 |
| T08 | Male | 40 | Middle school | Agricultural laborer | 3,001-4,000 | 5 | Stem family | 36 |
| T09 | Female | 32 | Bachelor’s degree | Unemployed | 3,001-4,000 | 4 | Stem family | 43 |
| T10 | Female | 30 | Associate degree | Kindergarten teacher | 4,001-5,000 | 4 | Stem family | 39 |
| T11 | Female | 37 | Master’s degree | High school teacher | 10,001-20,000 | 6 | Nuclear family | 48 |
| T12 | Female | 33 | Bachelor’s degree | Self-employed | 5,001-10,000 | 4 | Stem family | 41 |
| T13 | Female | 39 | High school | Unemployed | 2,001-3,000 | 5 | Stem family | 38 |
| T14 | Male | 35 | Associate degree | Self-employed | 4,001-5,000 | 4 | Stem family | 40 |
| T15 | Female | 31 | Bachelor’s degree | Primary school teacher | 5,001-10,000 | 3 | Stem family | 43 |
| T16 | Female | 41 | High school | Temporary worker | 2,001-3,000 | 6 | Stem family | 39 |
| T17 | Female | 34 | Associate degree | Self-employed | 4,001-5,000 | 5 | Stem family | 42 |
| T18 | Male | 36 | Bachelor’s degree | Training instructor | 5,001-10,000 | 4 | Stem family | 44 |
| T19 | Female | 28 | Vocational school | Unemployed | 2,001-3,000 | 3 | Stem family | 35 |
| T20 | Female | 35 | Bachelor’s degree | Kindergarten teacher | 5,001-10,000 | 5 | Nuclear family | 41 |

**Note.** A stem family refers to a household in which the parents and child live with at least one grandparent, whereas a nuclear family refers to a household consisting of the parents and child without a co-resident grandparent. Thus, family structure indicates grandparental co-residence rather than the presence or absence of grandparental involvement. Participants classified as living in nuclear families reported regular childcare involvement or childrearing support from non-co-resident grandparents. Monthly income is reported in RMB using the categories available in the source records.

**Table S3.** Nonzero edge weights of the estimated network

| **Node 1** | **Node 2** | **Edge weight** |
| --- | --- | --- |
| PP3 | PP4 | 0.729 |
| MP1 | MP2 | 0.612 |
| PI1 | PI2 | 0.374 |
| EC3 | EC4 | 0.361 |
| PI2 | PI3 | 0.327 |
| EC2 | EC3 | 0.322 |
| PI1 | PI3 | 0.320 |
| EC1 | EC3 | 0.307 |
| PD | MS | -0.264 |
| PP2 | PP4 | 0.219 |
| EC1 | EC4 | 0.209 |
| GS | MS | 0.204 |
| PP1 | PP2 | 0.203 |
| PD | PP2 | 0.198 |
| EC2 | EC4 | 0.178 |
| EC1 | EC2 | 0.162 |
| PP2 | PP3 | 0.149 |
| PD | SW | 0.124 |
| GS | MP2 | 0.123 |
| PD | PP4 | 0.122 |
| MP1 | PI3 | 0.121 |
| MS | SW | -0.121 |
| PP1 | PP4 | 0.118 |
| GS | MP1 | 0.115 |
| SES | PI3 | 0.109 |
| PP1 | SES | 0.102 |
| MS | SES | 0.101 |
| PP2 | SW | 0.100 |
| MP2 | PI3 | 0.093 |
| PD | SES | -0.090 |
| PP2 | MP2 | -0.088 |
| GS | PI1 | 0.078 |
| SES | MP1 | 0.075 |
| PP2 | PI1 | 0.074 |
| PP1 | EC4 | 0.068 |
| PD | GS | -0.066 |
| SW | MP2 | -0.059 |
| MS | PI2 | 0.058 |
| SES | PI1 | 0.057 |
| GS | EC2 | 0.055 |
| EC4 | MP2 | 0.055 |
| SW | PI2 | -0.055 |
| EC1 | PI3 | 0.053 |
| PP1 | MP1 | 0.052 |
| MS | PI3 | 0.050 |
| EC3 | PI3 | 0.044 |
| PD | MP2 | -0.040 |

**Note.** Edge weights are regularized partial correlation coefficients estimated from the Gaussian graphical model. PD = parental depression; PP1 = phone separation anxiety; PP2 = interpersonal conflict; PP3 = self-isolation; PP4 = problem recognition; GS = grandparent support; MS = marital satisfaction; SES = socioeconomic status; SW = child social withdrawal; EC1 = emotional understanding; EC2 = emotional regulation; EC3 = emotional expression; EC4 = emotional awareness; MP1 = mindful discipline; MP2 = present-moment attention; PI1 = home-kindergarten communication; PI2 = kindergarten-based involvement; PI3 = home-based involvement.

**Fig. S1** Stability of bridge expected influence

**Fig. S2** Stability of centrality indices

**Fig. S3** Edge-weight accuracy

**Fig. S4** Bootstrap difference test for edge weights

**Fig. S5** Bootstrap difference test for node strength

**Fig. S6** Bootstrap difference test for expected influence

**Fig. S7** Bootstrap difference test for bridge expected influence

**Note.** Black cells indicate statistically significant pairwise differences at the .05 level, gray cells indicate nonsignificant differences, and the values on the diagonal represent the original-sample estimates.

**Appendix C. Exploratory Sensitivity Analysis by Respondent Gender**

Methods. To examine whether the estimated network differed according to respondent gender, we conducted an exploratory independent-groups Network Comparison Test with 1,000 permutations, comparing female respondents (*n* = 3,237) and male respondents (*n* = 783). Because respondent gender was used to define the groups, the network variables were residualized within each group for parent age, child age, and child gender, but not respondent gender. Both group-specific networks were estimated using EBICglasso with *γ* = 0.50 and threshold = TRUE. The analysis compared overall network structure, global network strength, individual edge weights, and node-level strength, expected influence, and bridge expected influence. The Benjamini-Hochberg correction was applied to the edge-specific comparisons.

Results. The Network Comparison Test indicated no statistically significant difference in overall network structure between female and male respondents (*M* = 0.125, *p* = .880). Although the estimated global strength was 7.646 for female respondents and 6.291 for male respondents, this difference was not statistically significant (*S* = 1.355, *p* = .256). No individual edge differences remained significant after Benjamini-Hochberg correction (all adjusted *p* ≥ .611). Similarly, no significant group differences were found for node strength, expected influence, or bridge expected influence (all *p* ≥ .378). Thus, the primary network findings were broadly comparable across female and male respondents. Given the unequal group sizes, these findings should be interpreted as an exploratory sensitivity analysis rather than definitive evidence of gender invariance.

**List of abbreviations**

CES-D-10: 10-item Center for Epidemiologic Studies Depression Scale

CS: correlation-stability coefficient

EBICglasso: extended Bayesian information criterion graphical least absolute shrinkage and selection operator

EC: emotional competence

GGM: Gaussian graphical model

GS: grandparent support

MIPQ: Mindfulness in Parenting Questionnaire

MP: mindful parenting

MS: marital satisfaction

PD: parental depression

PI: parental involvement

PP: parental phubbing

SES: socioeconomic status

SW: child social withdrawal

VIF: variance inflation factor

References

Li, S. F., Wu, Y. L., Zhang, F. M., Xu, Q. Y., & Zhou, A. B. (2020). Self-affirmation buffering by the general public reduces anxiety levels during the COVID-19 epidemic. *Acta Psychologica Sinica*, *52*(7), 886-894. <https://doi.org/10.3724/SP.J.1041.2020.00886>

Ren, C. R. (2010). Measurement methodology on social economic status index of students. *Journal of Educational Studies*, *6*(5), 77-82. <https://doi.org/10.14082/j.cnki.1673-1298.2010.05.010>

Shi, B. G., & Shen, J. L. (2007). The relationships between family socioeconomic status, intelligence, intrinsic motivation and creativity. *Psychological Development and Education*, *23*(1), 30-34. <https://doi.org/10.16187/j.cnki.issn1001-4918.2007.01.006>
